# Designing Well-Being: A Qualitative Investigation of Young Patients’ Perspectives on the Material Hospital Environment

**DOI:** 10.1177/19375867231165763

**Published:** 2023-05-08

**Authors:** Shahin Payam, Jihad Hossaini, Katharina Zaschka, Anna Friedmann, Volker Mall

**Affiliations:** 1 Department of Sport and Health Sciences, Technical University of Munich, Germany; 2 TUM School of Medicine, Technical University of Munich, Germany

**Keywords:** hospital design, health facility environment, child health, disability, participatory research, arts-based methods

## Abstract

**Background:**

Physical surroundings of healthcare facilities are suggested to influence young patients’ well-being and hospitalization experiences.

**Purpose:**

The current research seeks to understand young patients’ views and perspectives of the hospital lobby and inpatient rooms. Thus, a qualitative study was carried out in a social pediatric clinic for young patients with disabilities, developmental delays, behavioral problems, and chronic health conditions, that is undergoing reconstruction.

**Method:**

Operating from a critical realist position, the study employed arts-based methods in conjunction with semi-structured interviews. The data were explored by employing thematic analysis.

**Results:**

37 young people between the age of four and 30 years participated in the study. The analysis illustrates that the built environment should contain comforting and joyful elements, while enabling patients’ autonomy. The ideal lobby was depicted as open and accessible and an ideal patient room as practical and adapted to personal needs.

**Conclusion:**

It is suggested that disabling and medicalized spatial arrangements and features may restrict young people’s sense of control and autonomy, while possibly posing a barrier to a health-promoting environment. Large and open spaces with comforting and distracting features are cherished by patients and may be embedded in a comprehensive, yet simple overall design and structural concept.

Most people in Western society visit healthcare facilities at some point in their lives. Our societies are constantly adapting to the complex requirements of modern public health. For instance, changing healthcare demands from acute to chronic illnesses in younger populations has led to rethinking practical standards in health-related fields (Perrin et al., 2014). In addition, theoretical advancements in health sciences shifted the focus from physical to mental and more complex conditions. This progress prompted the development of holistic care models. Concurrently, hospital designs have become relevant in maintaining patients’ well-being (Gaminiesfahani et al., 2020).

## Supportive Design and Psychosocial Effects of Hospitalization

Patients tend to characterize their associations with clinics negatively, reporting hospital fears (Andrade & Devlin, 2015). Specifically, children’s and adolescents’ hospitalization experiences can be demanding due to concerns related to pain and loss of autonomy (Bsiri-Moghaddam et al., 2011). Patients’ health is considered to be affected by design elements and spatial structures. For example, a lack of exposure to natural light or loud noises may increase stress and anxiety, cause headaches, and contribute to poor sleep quality (Schweitzer et al., 2004). Ulrich (1991) suggested that a supportive design helps patients handle the stress of hospitalization. A supportive design includes a sense of control, social support, and positive distractions (Andrade & Devlin, 2015). Enabling choices in the surrounding elements, such as lighting and entertainment systems, can provide a sense of control (Peditto et al., 2020). Positive distractions, including access to nature, may reduce stress and provide comfort (Ulrich, 1991). Several studies indicated that nature-related design elements (e.g., pictures of oceans, animal themes) and access to nature (e.g., gardens) are beneficial for patients’ healing processes (Gaminiesfahani et al., 2020). Options to connect with others, including a place for visitors and family members to sleep, promote perceived social support (Peditto et al., 2020).

***Patients’ health is considered to be affected by design elements and spatial structures.***

## Hospitalization Experiences of Patients With Long-Term Conditions

Young people with chronic conditions are often subject to hospital visits and stays. They have different hospitalization experiences compared to patients who are only admitted once that could harm their well-being (Pao et al., 2007). Children and adolescents usually share these experiences with family members (Watts et al., 2014). Their contentment can affect the parents’ emotional well-being (e.g., feelings of insecurity) and vice versa (Mayan et al., 2021). Consequently, this influences the treatment success of the therapy (Robinson, 1984).

The framing of a hospitalized young patient as “sick child” opposes normative cultural conceptualizations of childhood (James & Curtis, 2012), which is considered “a time of innocence, play and protection from the negative concerns of the adult world” (Crafter, 2018, p. 65). Thus, there are attempts to “normalize” children’s and young people with disabilities’ hospitalization experiences using different strategies (Anderson, 1981). Efforts of normalization could be palpable in the material surroundings of hospitals, where leisure activities are enabled in different spaces. Playrooms, for instance, evoke a sense of familiarity in the foreign environment (Crafter, 2018; James & Curtis, 2012; McLaughlan et al., 2019; Robinson, 1984).

## Environmental Preferences in Different Developmental Stages

Previous research examined the effects of physical surroundings on diverse age groups and patients with various conditions (Eisen et al., 2008). As individuals in different developmental stages have specific needs and abilities, their preferences regarding designs and material environments vary. Children with physical disabilities need accessible playgrounds, while people with visual impairments need landmarks for orientation (Courtney & Keith, 2017). Following Piaget’s theory of cognitive development (Piaget, 1964), researchers highlighted differences in design preferences between infants, toddlers, and early and later adolescents (Cartland et al., 2018; Eisen et al., 2008). Younger children, for example, need space for play, while adolescents rather have space for socializing with others (Cho et al., 2019; Peditto et al., 2020). Indeed, hospitalized teenagers, who are in a transitional period to adulthood, expressed a dislike for childlike environments (McLaughlan & Willis, 2021; Ullán et al., 2012). Whether this holds up for children, adolescents, and adults with multiple disabilities is yet to be determined. In light of developmental differences of patients, designers and researchers need to carefully consider stakeholders’ perspectives when reconstructing healthcare facilities.

## Significance

To our knowledge, no study dealing with the clinical environment of people with disabilities in different age groups was conducted in Germany. Earlier research explored the topic predominantly in Commonwealth countries and the United States of America. Based on a universal multipayer healthcare system (including public and private health insurance) offering free healthcare for all, German hospitals are largely publicly funded. Both public and private hospitals are accessible to anyone, allowing for the inclusion of people with a variety of backgrounds.

Following the United Nations Convention on the Rights of Children (1989, Articles 3 and 18) and the United Nations Convention on the Rights of Persons with Disabilities (2008, Articles 4 and 31), it is considered paramount to include the voices of vulnerable groups in research and decision-making processes. Accordingly, study designs and research methods need to consider patients’ abilities and needs. Depending on how developed participants’ communication skills are, for instance, data collection methods need to be adjusted. Younger children may want to draw ideas, because they are unable to express their feelings verbally. Teenagers are cognitively further developed and can discuss more abstract concepts. Therefore, they may be more inclined to speak to interviewers. Thus, different creative methods, such as arts-based research, were put into practice (Angell et al., 2015). Arts-based methods can simplify the dialogue with children or people with disabilities, who are underrepresented in research (Boydell et al., 2012), despite often constituting the target group (e.g., in a children’s clinic). Such methods can empower participants to voice their perspectives, while using familiar and comfortable communication practices (Boydell et al., 2012).

## Context and Aim

In view of this, a qualitative study was conducted in a social pediatric clinic for young patients with disabilities, developmental delays, behavioral problems, and chronic health conditions in Germany, which is undergoing reconstruction. Details on the clinic are summarized in Table 1.

**Table 1.** Setting—Social Paediatric Centre.

Location	▪ kbo-Kinderzentrum München-Großhadern, Munich, Germany
Year of construction	▪ 1989
Specialty	▪ Social pediatrics: social integration of children with disabilities, focusing on family, kindergarten, and school contexts
Target group	▪ Children and adolescents with disabilities between 0 and 18 years old ▪ Adults with severe or multiple disabilities up to around 30 years old
Goal	▪ Enable patients to participate actively and in a self-determined manner in everyday life
Therapeutic focus points	▪ Motor skills, cognition, speech, hearing, behavior
Services	▪ Counseling and diagnosis ▪ Physical, psychological, occupational, and speech therapy ▪ Complementary treatment (e.g., music therapy, animal-assisted therapy)
Operational structure	▪ Outpatient department treating around 13,000 families per year ▪ Two inpatient units treating around 900 patients per year. Depending on treatment goal, children or families stay between 2 and 6 weeks at the inpatient department
Number of beds	▪ Approximately 50
Start of reconstruction	▪ 2018
Anticipated end of the reconstruction	▪ 2023
Reasons for reconstruction	▪ Spaces cannot keep up with recent healthcare demands ▪ Lack of space, the new facility will be equipped with more rooms for therapy

This investigation aims to understand the patients’ views on their material surroundings to implement them into the new clinic design. The following questions are sought to be answered: How do young patients describe and talk about the clinic lobby and the hospital bedroom? What are the implications of their descriptions?

## Method

### Ethical Considerations

Ethical approval was granted by the ethics commission of the TUM School of Medicine in September 2019 (394/19 S-SR), and investigators signed nondisclosure agreements. Parents of minors and patients of legal age (above 18 years) received participant information sheets, as well as consent forms after being verbally informed, and only if their child expressed an interest to learn more about the investigation. Moreover, participants were handed age-appropriate assent/consent forms. Children under 13 years received participant information sheets with simplified explanations of the study on which they could declare their consent. Children 13 years and older received similar sheets with more detailed information. Every participant received a pseudonym to avoid a direct connection between the data and the individual.

### Research Team and Reflexivity

Two women and three men were part of the team. All but the third author have experience working with children in clinical, therapeutic, and educational settings. The primary author is a practicing child psychologist and researcher, who has previously worked on studies related to hospital designs. The second and the third author have close relatives with a disability, thus providing them with more of an insider’s view. The last two authors are researchers at the children’s center.

### Data Collection

Data were collected from individuals based on certain criteria that are detailed in Table 2.

**Table 2.** Eligibility and Recruitment.

	Description	Details
Sampling	▪ Purposive variation sampling	▪ The clinic’s clientele is heterogeneous (see Table 1). The aim was to include a wide variety of views.
Inclusion criteria	▪ Patients or their siblings from the outpatients or inpatients services who were at least 4 years of age ▪ Potential participants needed to understand either German, English, or Arabic in accordance with the researchers’ language skills ▪ Patients needed to be able to communicate in any form (verbally, nonverbally, with assisting devices) and assent/consent to participation	▪ Previous research has shown that children above the age of 4 years are capable of communicating their perspectives on material environments (Gaminiesfahani et al., 2020). ▪ The center also treats adult patients who have developmental delays due to severe disabilities and require therapy beyond the age of 18 years. In accordance with our sampling approach, we did not set an upper age limit to capture the perspectives of all patients.
Exclusion criteria	▪ Patients with disabilities that prevented them from communicating and assenting	
Recruitment	▪ Face-to-face ▪ Flyers—handed to parents by the staff on the day of admission or by the researchers themselves ▪ Two researchers actively approached parents with their children in the entire clinic	▪ Flyers included information about the studies, and pictures and contact information of the two investigators. On the back of the flyer, parents could indicate if they or their children wished to receive more information about the study by ticking a box and writing down their last names, before handing the flyers back to the staff. ▪ Flyers were hung up in the inpatient units and the hospital lobby

A man (second author) and a woman (third author) collected data using arts-based research techniques. The current investigation utilized the “draw, write and tell” method (Angell et al., 2015), where participants could choose between drawing a picture or writing a letter about the lobby or the model room. If the patients decided to do so, they received a booklet, in which they could write down their artist names (pseudonyms) and their age on one page, and express their artistry on the other. Afterward, patients took part in a semi-structured interview, using the letter or drawing as the center of the conversation. During the interview, patients were asked questions about experiences and perspectives on the lobby or patients’ rooms. Every interview was recorded, and we asked every participant for permission to take a photo of their art pieces, so that they could keep the original. The outcomes of the data collection process are summarized in Table 3.

**Table 3.** Data Collection Process.

Data collection period	▪ 11/2019 to 06/2021
Topics	▪ Model room (*N* = 30)
	▪ Lobby (*N* = 20)
Arts-based methods ^a^	▪ Drawings (*N* = 16)
	▪ Letters (*N* = 5)
Interview duration (minutes)	▪ *M* = 19.162
	▪ *SD* = 12.833
	▪ Ra = 4–60
Presence of third persons	▪ Parents were present in 19 of the interviews (e.g., to facilitate communication for participants with severe speech impairment)
Field notes	▪ were noted down after the interview (e.g., interruptions)
Saturation	▪ Data collection and analysis were conducted simultaneously until data saturation was reached

*Note*. Fifty interviews were conducted. *M* = mean; *SD* = standard deviation; Ra = range.

^a^ One patient wrote a letter and drew a picture.

**Figure 1.**
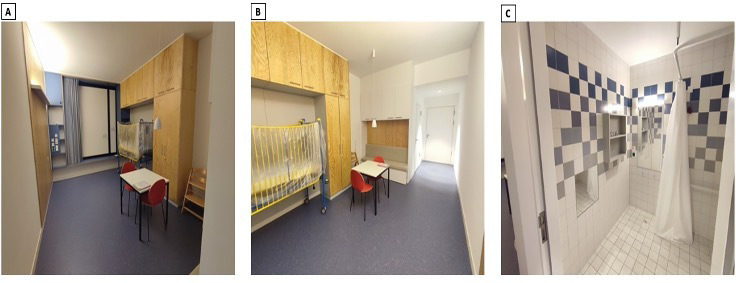
The model room.

More than half of the patients did not want to write or draw and preferred just being interviewed, giving two primary reasons. Firstly, they (especially patients aged above 12 years) wanted to be seen as independent grown-ups, as opposed to vulnerable children. Secondly, the physical disabilities along with limitations in motor skills prevented them from writing or drawing. Patients could choose the place for the conversations to enable their sense of autonomy. Therefore, interviews concerning the entrance hall were conducted, for example, in the lobby or the cafeteria. Interviews about the patients’ rooms were mostly conducted in a model room. This room was provided by the construction managers and served as an example of what a finished patient bedroom in the future clinic might look like (see Figure 1). Participants were given the option to ask for study results.

### Participants

Overall, 37 patients took part in the study (see Table 4), whereby 13 of them talked to us about both topics, the lobby, and the patients’ room.

**Table 4.** Participants’ Characteristics.

Characteristics/Age	4–6 Years	7–12 Years	13–17 Years	18 Years and Older	*Total*
*Sex*	*N*				*N (%)*
Boys/men	2	9	8	3	22 (59)
Girls/women	3	3	6	3	15 (41)
*Disability*					
Physical ^a^	2	6	8	2	18 (49)
Speech ^b^	3	3	1	0	7 (19)
Multiple ^c^	0	3	5	4	12 (32)

*Note*. *N* = 37. Participants were on average 13.5 years old (*SD* = 6.3).

^a^ Participants who were restricted in moving one or multiple body parts due to loss or limitation.

^b^ Participants with speech impairments that ranged from mild stuttering to great difficulty communicating and required the use of a speech computer.

^c^ Participants who had more than one type of impairment (e.g., participants with intellectual disability and spina bifida).

### Data Analysis

The data were analyzed within a critical realist framework, employing a reflexive thematic analysis (Braun & Clarke, 2019). Critical realism assumes an independent reality outside of human perception and construction (Pilgrim, 2019). However, critical realists believe that there are different domains of reality and that any form of experienced truth is associated with theory. This assumption makes human knowledge potentially “always fallible” and its relevance dependent upon the circumstances (Danermark et al., 2002). Subsequently, the current study focuses on explanation rather than description of expressions (see Maxwell, 2012). The material hospital environment is the subject of inquiry. In accordance with Campbell et al.’s (2021) approach to applied health research, attention was given to semantic and latent meanings. An inductive approach was used to determine mainly participant-driven themes and to avoid potential constraints of previous theories. The analysis of drawings, letters, and transcripts followed Braun and Clarke’s (2019) six steps presented in Table 5.

**Table 5.** Data Analysis—Thematic Analysis following Braun and Clarke (2019).

Stage	Process	Contributors
Transcription	▪ Recordings were transcribed verbatim ▪ Included: Verbal utterances, occasional background sounds, laughs, coughs, nonverbal sounds, and signs ▪ Pauses noted down by dots ▪ Inaudible phrases or words indicated by question marks ▪ Terms in square brackets signified words that were added by an author ▪ Three dots in square brackets indicated omitted words or phrases	The second and third authors transcribed the interviews. Outlined data extracts were translated by the second author and checked for accuracy by the first, who is a native speaker in both German and English.
Data familiarization	Transcripts were actively read while writing down first thoughts	The second author read the transcripts, determined initial codes, and developed themes.
Coding	Coding involved a systematic search for meaning patterns in interview transcripts, letters, and pictures	
Theme development	Themes were developed through reorganizing semantic codes, while looking for differences and similarities in meanings	
Theme refinement	Themes were reviewed in context with interview transcripts, letters, and pictures and in context with codes	Themes were reviewed, defined, and named by the lead and the second author.
Theme definition	Single themes were finalized and put in context with other themes	
Reporting	The report was finalized, and themes were slightly changed	

*Note*. The six steps were repeated back and forth until a coherent report was produced.

## Findings

In general, patients described an optimal hospital environment as joyful, helpful, and as an open space, containing clear and simple structures. Taking on agentic and parental roles, patients were oriented toward practical aspects while presenting intricate solutions for the overall design concept and particular spatial elements. Three themes were determined and are presented in the following subsections.

***Taking on agentic and parental roles, patients were oriented toward practical aspects while presenting intricate solutions for the overall design concept and particular spatial elements***

### Not a (Typical) Hospital Environment

**Figure 2.**
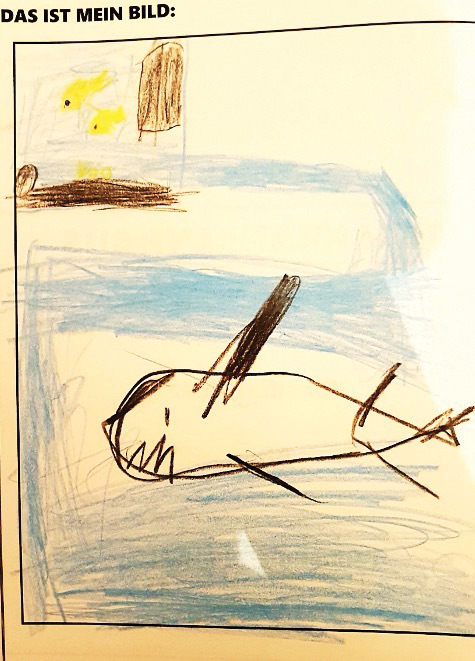
Drawing by Bragi (Boy, 10 years).

Familiar, distracting, and entertaining surrounding elements were considered important for the entire children’s center. Older participants (of legal age) with delays in cognitive development wished for play spaces, similar to children and early adolescents in the sample. Indeed, participants in all age groups positively acknowledged distracting design features in the current lobby and expressed further wishes. One participant drew a picture about his wishes (see Figure 2).

The top left of his picture shows the current aquarium and a shark surrounded by water is present in the center. Bragi explained that the floor of the lobby needs to be like an aquarium itself, with sharks in it, and suggested other animals in the lobby. These are reminders of nature that contrast a sterile clinic, highlighting the broader idea of a space that does not look like a “typical” hospital lobby. Indeed, the space should feature living elements, which potentially promote wellness (Ulrich, 1991). However, though the aquarium was viewed positively, it must be incorporated in a way that enables free movement and lessens barriers. All age groups, particularly wheelchair users, shared this sentiment. Moreover, the lobby environment should distance itself from the traditional symbols of medical institutions. Instead, it may resemble a natural setting with healthy living beings associated with leisure activities. This is further highlighted by Nanna, as she talks about the bedroom:

Nanna: Then, I don’t know, an elf room, a pirate room (Int: Mh-mh, right) I think in the Europa-Park [theme park in Germany], there’s a room like that—one more pirate-like (Int: Mh-mh) You can find that in Legoland, these amusement parks, there’s always something like that in hotels (Int: Mh-mh) So if it’s like this, somehow like this…the sickbed also more like this- with wood somehow (Int: Aha, okay) Like a pirate bed or a princess bed…and this window sill that is just a bit…wider, so that you can sit on it better (Int: Mh-mh) And also with cushions and somehow a nice…colorful curtain, matching the theme of the room. (Int: Mh-mh) And somehow also on the wall graffiti or something beautifully painted…uh…elf forest, unicorn…exactly, somehow like that. (Int: Mh-mh). (Girl, 16 years; Translation)

Here, Nanna details different colorful, thematically arranged rooms and compares the bedrooms to those in amusement parks and hotels. Despite previous research proposing that adolescents are opposed to child-like environments (Ullán et al., 2012), our older participants (12 years and above) stressed the importance of child-friendly and playful designs. In line with the descriptions of the lobby, bedroom features (“princess bed”) are contrasted with clinic elements (“sickbed”). This indicates a dislike of medicalized features and spaces, which can constrict young people’s sense of control and independence (Crafter et al., 2015; Gaminiesfahani et al., 2020; James & Curtis, 2012; Kanyeredzi et al., 2019; McGrath & Reavey, 2018; Robinson, 1984). In line with past findings (Gaminiesfahani et al., 2020), these results highlight the notion that designs “normalize” the experience by creating a more familiar, comfortable environment. Moreover, patients suggested that well-being in the patients’ room is associated with feeling like being in a hotel:

Sophia: [Picture] of…maybe a landscape or something (Int: Mh-mh) Then it would look—(Int: And- hm?) Ah. Then it would look more like a hotel room. (Int: Ah) And not as it would be a hospital. Otherwise, you’d feel sick (laughing). (Girl, 16 years; Translation)

Sophia would like to see a decorative element associated with a hotel, rather than a hospital room that (re)produces feelings of illness, demonstrating the potential impact of the environment on the individual’s emotional and physical state. With these modifications, she may be seeking to redefine the meaning of the space into something more comfortable and enjoyable (Crafter et al., 2015)—in this case a holiday, which can evoke a sense of being a visitor or tourist, rather than a patient.

***Moreover, the lobby environment should distance itself from the traditional symbols of medical institutions.***

The sterile clinic character was not viewed in a neutral fashion, but rather as a pathologizing environment that adversely impacts the mental state. Therefore, spatial modifications to counteract the typical hospital associations do not only serve the purpose of increasing patients’ well-being, but also prevent further negative health consequences. While a medicalized space might pathologize patients and threaten their independence (Kanyeredzi et al., 2019), recreational spaces, enabling children to have fun, support a sense of control (e.g., Adams et al., 2010; Lambert et al., 2014b). Material surroundings are viewed as part of cultural or organizational contexts. When considering hospitals as physical, social, and symbolic spaces (e.g., Koller & McLaren, 2014), a medicalized space may inherently position the patient in a constricting inferior role. Consequently, a patient may surrender autonomy and independence to medical authority (Kanyeredzi et al., 2019; McGrath & Reavey, 2018). A sense of control could be further threatened by complex and confusing spatial arrangements, which were often discussed in the context of the lobby as shown in the next theme.

### An Overwhelming and Confined Space

The lobby was depicted as an unclear, overwhelming, and confusing space. All patients above the age of 6 years raised wayfinding issues, while portraying it as a crowded place and pointing out restricted pathways. Issues of navigation were presented:

Fulla: So, I find it [the lobby] a bit unclear […] so one does not know where something is if you have not been here often […] there are a lot of corners and doors an—(Int: Mhm.) you—you do not directly see where to go […]. (Girl, 16 years; Translation)

Fulla explains how the lobby layout seems confusing, particularly if someone is new to the place. This portrayal of patients as vulnerable strangers navigating through a complex environment works to construe the experience in the children’s center as an uncertain one (Coyne, 2006). Therefore, one patient wrote a letter expressing wishes to create more openness and facilitate orientation and arrival:

Dear Prof. Mall,

an entrance area with a clear map on each floor. The information is right at the entrance. I like that. The doors are electric. I think it’s good that you can see which doctor is on duty today. I would like to have a place where you can leave your luggage in the meantime and parking spaces that are relatively close to the entrance and where you can quickly bring your luggage into the building. I would like there to be Wi-Fi and internet, I would like to feel at home in the children’s center, the play area should be accessible for wheelchairs and there should be games for different age groups. I would like the entrance area to have no steps, not be small and have a wider foyer. I want toilets that are accessible for wheelchairs with changing table.

Kind regards from Erdbeere. (Boy, 10 years; Letter translation)

Erdbeere’s letter conveys a wish for accessible and larger spaces, as well as the need for a place to store luggage. While arguing for a simple arrival and useful structures in the lobby, he contrasts a “small” area to a “wide foyer,” which opposes confining structures and advocates for more open space. Erdbeere extends this space to the outside parking area, illustrating how the overall hospital experience stretches beyond the treatment location. Participants’ functional evaluation of the physical environment and their desire for practical solutions in the lobby and the bedroom are noteworthy, considering critiques that healthcare environments aim more toward utilitarian than person-centered spaces (Koller & McLaren, 2014). Notions of accessibility (e.g., automatic doors) and openness are important considerations for perceived freedom of movement and orientation (Adams et al., 2010). This might offer young patients a sense of control and independence (Lambert et al., 2014a; Ulrich, 1991)—an aspect that may already be challenged in the medicalized space (Kanyeredzi et al., 2019). For our participants above the age of 6, autonomy and control could relate to being able to move through the building independently. Currently, certain surrounding features are perceived as disabling, thus, an open room was described as more enjoyable:

Eira: [The most important thing about the lobby is] […] that it is clear […] that I know as quickly as possible where I have to go […] that it is bright…. and that it is simply appealing, and I don’t have a feeling like “Oh great…. uh…Farewell, beautiful world,” like that […] Yes. That it’s really an open, pleasant room. You feel already miserable when you’re a patient…. Also, not too low, not too dark, because that’s always constricting (Girl, legal age; Translation).

Eira contrasts a well-lit, inviting environment and a constricting, uninviting place to illustrate the difference between the often restricting hospital environment and the outside world. She highlights the importance of such a space in particular for the emotional state of someone who is suffering, namely a patient. More specifically, Eira points out how an already demanding hospitalization experience could be worsened by confining spatial features. This contrast to the “beautiful world,” essentially works to frame the hospital as a place that constricts a sense of freedom and control (James & Curtis, 2012; Peeters et al., 2018). Within this theme, there was a tendency for older patients (over 12 years) to describe emotions of being overwhelmed, while younger patients were more likely to point out individual constricting elements or places. However, there were exceptions to this among older participants (over 16 years) with multiple disabilities and cognitive impairments, who were less concerned with abstract connections between emotions and spatial elements.

Nonetheless, especially patients over 12 years mentioned that a larger space might relieve the pressure of being in a hospital (Bsiri-Moghaddam et al., 2011). This allows for more breathing room, which might support a sense of freedom and control (Lambert et al., 2014a). Correspondingly, having control over spatial elements was also considered important for the bedrooms as outlined next.

### A Practical Room to Fit Everyone’s Needs

More than half of the participants across all age groups described the importance of usefulness, simple handling of furniture, aspects of accessibility, and options for personalization in the model room. Patients expressed that a hospital bedroom needs to be functional and suitable to individual needs by proposing changes to material elements:

Eira: Further the sink is quite low, that’s clear. That was thought for children, that it is a bit lower. But maybe it would not be bad if one could somehow have the possibility to do something with the height, because if then again now an adolescent came, the sink would be at his knee height. That’s a bit impractical. […] It would not be bad. If then just different age groups would be in these rooms. (Girl, legal age; Translation)

Arguing for equal access, Eira noticed the low sink in the bathroom and stated individualized options to be more practical. She makes a case for age-appropriate solutions in material environments (Lambert et al., 2014b), illustrating the importance of accessibility and suitability of physical surroundings. Such a statement underlines the relevance of tailoring spaces to a wide range of individual needs (Freund, 2001). Indeed, material elements (e.g., unreachable cabinets) were often mentioned by the participants as being disabling (Freund, 2001) and counterproductive to their sense of independence (James & Curtis, 2012). It must be considered that a major goal patients pursue in this clinic is self-determination. Hence, our participants may illustrate a greater awareness of material elements that challenge their independence. Other patients raised issues of accessibility and usefulness on behalf of their parents:

Wali: I don’t think that’s right. (Int: OK, how-wa-) Because opening the bed every time is a pain in the back for the parents. […] The better solution is to have a bed with a remote control (Int: Mhm.) so that the parents also have a remote control. (Int: OK.) And that you—that you make sure that it runs automatically like the other things, like the doors (Int: OK.) that you also have automatic buttons (Int: Mhm.) and that the bed opens directly with a button (Int: Mhm.) […]. (Boy, legal age; Translation)

While arguing to facilitate the use of the furniture pieces, Wali is taking responsibility for the well-being of his parents, thus taking on the parental role. Patients’ contentment is potentially related to parents’ sense of convenience and/or well-being during their stay, as hospitalizations of young patients are often shared experiences with family members (Watts et al., 2014). Moreover, remote solutions could enable access to physical elements, which might support a sense of control for children in foreign environments (Lambert et al., 2014b) and participation for young people with disabilities (Biklen, 2000; Freund, 2001). The notion of independence was consistent among all age groups, including and particularly among wheelchair users below the age of 12 years. This possibly relates to the fact that (younger) wheelchair users were highly aware of any material restrictions in their surroundings and described alternatives. Due to their physical disability, they may be more perceptive to barriers in the environment. According to Freya, control over the material environment could also be achieved through personalization options:

Freya: I think I would [at the empty white wall] just um…so, a cabinet, I wouldn’t put a cabinet there. I’d rather put a thin shelf that maybe runs along the…at belly or chest level, um, and either…. I think it would be- I’d put it along the whole…. I think it would be- I would do it along the whole corridor until you get to the room and then it stops when the room actually starts, so that you also have a separation between the corridor and the room (Int: Ah) Um, and then I would just put personal things there, either pictures or cards or…. Posters theoretically […]. (Girl, legal age; Translation)

Freya argues for a shelf with multiple purposes, which usefully divides up the room and serves as a customizable space. Such options offer the possibility of reorganizing an unknown space into a homely and familiar environment (James & Curtis, 2012). Patients in all age groups indicated that age-appropriate or personal elements and the practical aspects of the space are important issues. This underlines the value of age-appropriate designs and activity options, thereby echoing previous research (Gaminiesfahani et al., 2020; Lambert et al., 2014b; Peeters et al., 2018; Ulrich, 1991). Since participants value functionality and accessibility, age-appropriateness arguably goes beyond visual layouts and recreational spaces for children, as it seems that they wish to manage their daily tasks by themselves and according to their abilities.

### Implications for Research

Patients with multiple and severe disabilities in different age groups are competent and helpful research participants who can detail their wishes regarding built environments. Indeed, they consider complex and multiple perspectives, such as economic, emotional, and social aspects. Challenges in data collection with participatory arts-based methods, specifically for young people with physical impairments may be overcome by allowing the participants to choose communication options they feel most comfortable with. In this regard, future investigations could, for instance, inspect or provide different creative methods that enable the artistic expression of people with motor impairments.

Our findings indicate that age differences in environmental preferences are not as clear-cut as previously presented (Peditto et al., 2020; Ullán et al., 2012), especially when adding the disability dimension to the analysis. We have shown that in a sample that is heterogeneous in age and ability, there is a great deal of consistency within the views on positive distraction and control. Even though different age groups emphasize different material aspects, there is a consensus around personal suitability, accessibility, and functionality. Nonetheless, further research is needed on differences in spatial preferences across different types of disabilities and different age groups.

### Implications for Practice

Large and open spaces with comforting and distracting features are cherished by patients and may be embedded in a comprehensive but simple overall concept. Every aspect of the environment needs to be tailored to a wide range of abilities (Freund, 2001), ages, and service users. Providing young patients with such an environment can facilitate their growth in different developmental stages. We recommend practical surroundings that facilitate the use for families, and enable patients’ freedom of movement and choice, while providing appropriate options to pass time. Thematically coherent environments resembling homes, hotels, or places of familiarity and fun are regarded as comforting and welcoming. Conversely, physically limited spaces and a sterile clinical atmosphere potentially challenge patients’ autonomy, while possibly hindering health promotion by increasing distress (McGrath & Reavey, 2018). A health-promoting environment may not only be tailored to young patients, but also to parents, as the children and young adults seem to be mindful of their parents’ experiences and emotions during their hospital visit.

### Limitations

The results are limited in generalizability, as they represent the perspectives of 37 young people, who were patients at a children’s social pediatric clinic in southern Germany. Nonetheless, the study extends previous findings through views from a hard-to-reach German population. Furthermore, since almost no personal information was collected to protect participants’ anonymity, conclusions cannot be drawn about potential differences in opinion based on, for example, socioeconomic status or cultural background. Lastly, a health-promoting environment consists of a larger concept including hallways, therapy rooms and other clinical areas. However, despite patients’ occasional mentioning of other spaces and rooms in the children’s center, the findings are specific to descriptions of the lobby and clinic bedrooms.

## Implications for Practice

- Hospital spaces for children/young people with disabilities should be large and open and contain comforting and distracting features resembling homes, hotels, and places of fun.
- The environment should accommodate a wide range of abilities and ages, such as wheelchair users, adults with intellectual disabilities and children with vision impairment.
- Surroundings need to be practical and include remote solutions for furniture and doors to facilitate the use for families, enabling freedom of movement.
- Health facilities should provide a choice of entertainment options and decorative elements for patients of all age groups.
- A health-promoting environment should be tailored to young patients, as well as parents.

## Supplemental Material

Supplemental Material, sj-pdf-1-her-10.1177_19375867231165763 - Designing Well-Being: A Qualitative Investigation of Young Patients’ Perspectives on the Material Hospital Environment Click here for additional data file. Supplemental Material, sj-pdf-1-her-10.1177_19375867231165763 for Designing Well-Being: A Qualitative Investigation of Young Patients’ Perspectives on the Material Hospital Environment by Shahin Payam, Jihad Hossaini, Katharina Zaschka, Anna Friedmann and Volker Mall in HERD: Health Environments Research & Design Journal
