# Supplementary material for: Designing Well-Being: A Qualitative Investigation of Young Patients’ Perspectives on the Material Hospital Environment
Source: HERD. 2023 May 8;16(3):168–81. doi: 10.1177/19375867231165763 (PMC10328141; doi:10.1177/19375867231165763)
Supplement: Supplemental Material, sj-pdf-1-her-10.1177_19375867231165763 - Designing Well-Being: A Qualitative Investigation of Young Patients’ Perspectives on the Material Hospital Environment [file sj-pdf-1-her-10.1177_19375867231165763.pdf]

Designing well-being: A qualitative investigation of young patients' perspectives on the material hospital environment

Additional File 1

Interview Questions

Lobby

- How do you like the lobby?
- What would you change here?
- What do you think about the furniture and decoration? (doors, lighting, walls)
- What would you like to see in the lobby?
- What is something you'd definitely not want in an entrance hall?
- If there was only one thing you could change, what would it be?
- How do you feel when you come in?
- Do you know directly where to go when you enter the lobby?

Bedroom

- How do you like this room?
- Do you find anything particularly nice here?
- What do you think about the furniture and decoration? (Furniture, windows, doors)
- What do you not like at all?
- What would you like to have/see in this room?
- If there was only one thing you could change, what would it be?
- How would you design this wall?
